# One-step purification of a bioactive PAK1-derived peptide

**DOI:** 10.1016/j.ab.2025.116014

**Published:** 2025-11-19

**Authors:** Djamali Muhoza, Emily P. Esquivel, Stacy R. Hunter, Patience S. Okoto, Thallapuranam K.S. Kumar, Paul D. Adams

**Affiliations:** a School of Math and Natural Sciences, University of Arkansas at Monticello, Monticello, AR, 71655, USA; b Department of Chemistry and Biochemistry, University of Arkansas, Fayetteville, AR, 72701, USA

## Abstract

The serine/threonine kinase PAK1 serves as a mediator of cytoskeletal reorganization and cancer-related signaling downstream of the small GTPases. Due to the challenges in purifying PAK1 complexes, a 46-residue peptide from PAK1, is widely used to study PAK1-Cdc42 signaling. Traditionally, this purification involved multi-step chromatography of recombinant GST-PBD46 complexes, yielding approximately 1 mg per 1.5 L culture. In this study, a 30 min heat treatment step after thrombin cleavage was used to precipitate GST while leaving pure PBD46 in solution. This step eliminated the need for further affinity and size-exclusion chromatography steps. This improved protocol produces proteins with a 6.5-fold higher yield, halves the purification processing time, and produces peptides with ≥95 % purity. Mass spectrometry, CD, fluorescence, and ^1^H─^15^N HSQC NMR confirmed the heat-purified PBD46’s identity, structure, folding, and binding to Cdc42. The method also successfully separated other small peptides (e.g., ACK1) but not larger folded proteins. This rapid and scalable approach facilitates peptide production and biochemical studies without compromising the structural or functional integrity. We believe that the method described herein is applicable to other stable GST-fused recombinant proteins.

P21-activated kinases (PAKs) are effector kinases of the Rho-family GTPases that regulate the cytoskeleton, gene expression, and cell survival [1]. Group I PAKs (PAK1-3) are activated by the small GTPases Cdc42 and Rac1 [2]. In many cancers (e.g., breast and colorectal), PAK1 is hyper-activated and correlates with tumor progression [3-5], making PAK1 an important therapeutic target [6]. To study PAK1-Cdc42 signaling, researchers isolated the PAK1 CRIB domain (residues 67–112) as a 46-residue peptide (PBD46) [7]. PBD46 binds Cdc42 with nanomolar affinity and mimics PAK1’s binding interface [7]. Traditionally, GST-PBD46 is expressed in E. coli and purified by multi-step chromatography: one glutathione affinity step to capture GST-PBD46, thrombin cleavage to release PBD46, a second affinity capture to remove GST, and size-exclusion chromatography to further purify the sample (Fig. 1). This process produces a pure peptide but requires ~4–7 days and large culture volumes to obtain sufficient material (typically ~1 mg PBD46 from 1.5 L of cells).

To increase the protein yield and decrease the purification time, we have developed a new method using the peptide’s heat stability. Recently, similar heat-precipitation methods have been used to isolate other small heat-stable proteins and peptides [9,10]. After affinity purification and thrombin digestion, the protein solution was heated to 50 °C for 30 min and then centrifuged twice at 16,200×*g* for 10 min. Heat denatures and subsequently aggregates GST (~26 kDa) while leaving the small Ras-related effector peptide, PBD46 (~5 kDa; which migrates as a ~10 kDa dimer [8]) in the supernatant. As shown by SDS-PAGE (Fig. 2A), heating to 50 °C aggregates GST, yielding a pure PBD46 peptide after centrifugation. Applying this single heat step to 1.5 L of culture produced ~6.5 mg of PBD46 (a 6.5-fold increase in yield) and roughly halved the total purification time (Fig. 2B). Mass spectrometry also confirmed the identity of these peptides. LC-MS of the heat-treated sample showed a major species at 10.23 kDa, indicating the presence of a non-covalent, PBD46 dimer [8]. MALDI-TOF analysis of tryptic fragments yielded peptides matching the PAK1 67–112 sequence [11]. Thus, the heat-purified product was the correct 46-amino-acid PAK1 fragment. Notably, SDS-PAGE indicated that the heat-purified peptide was at least as pure as the conventional sample, and centrifugation efficiently separated all GST from the peptide solution (Fig. 2A).

We then tested whether heat purification affected the structure or function. Circular dichroism spectra (190–250 nm) of the heat-purified and conventionally purified peptides were identical (Fig. 3B), indicating no change in the secondary structure [12]. Intrinsic tryptophan fluorescence (300–400 nm) of the two peptides was also similar (Fig. 3A), indicating that the peptides’ folding is unaffected. The heat purified peptide showed the same emission profile with slightly lower intensity; likely due to a minor difference in concentration. We assessed binding by labeling Cdc42 with an extrinsic fluorophore (sNBD) and titrating PBD46 [13]. Titration curves for heat-treated and conventional PBD46 were identical, yielding K_d_ ≈ 83 nM for the heat-purified peptide (Fig. 3D), consistent with previously reported affinity [14]. Finally, 2D ^1^H─^15^N HSQC NMR spectra of uniformly ^15^N-labeled PBD46 were overlaid: the peaks matched between heat-treated and conventional samples (Fig. 3E), indicating identical backbone conformation. Together, these assays confirmed that heat purification preserved the peptide’s sequence, structure, and activity.

To demonstrate its effectiveness, we applied the heat protocol to other GST-fusion proteins. Fig. 4A shows SDS-PAGE of a GST-ACK1 fusion protein (ACK1 is a Cdc42-binding, ACK kinase-derived peptide) after heating: the small ACK1 peptide (~6 kDa) remained in the supernatant while GST was pelleted. In contrast, heating GST-Cdc42 (21 kDa) caused almost all of the proteins to aggregate (Fig. 4B). Thus, the one-step heat separation effectively purifies only small, heat-stable peptides, not larger folded proteins [9]. This method can be successfully used to isolate pure Cdc42-binding peptides such as components of ACK1, Wasp, and Pak4 [15,16].

In summary, we present a one-step heat-treatment method that greatly simplifies the purification of the PAK1-derived PBD46 peptide. This protocol produces a pure peptide with >6-fold higher yield and in about half the time of the standard chromatography-based procedures. Importantly, the heat-purified PBD46 is native in structure and function. This approach should facilitate biochemical and structural studies of PAK1 and Ras-family GTPase interactions and is readily scalable for mass peptide production. Its utility is further supported by the growing interest in bioactive peptides for therapeutics and research [17,18]. The method presented here is applicable to large-scale purification of structurally stable, heat tolerant-fused peptides.

## Figures and Tables

**Fig. 1.**
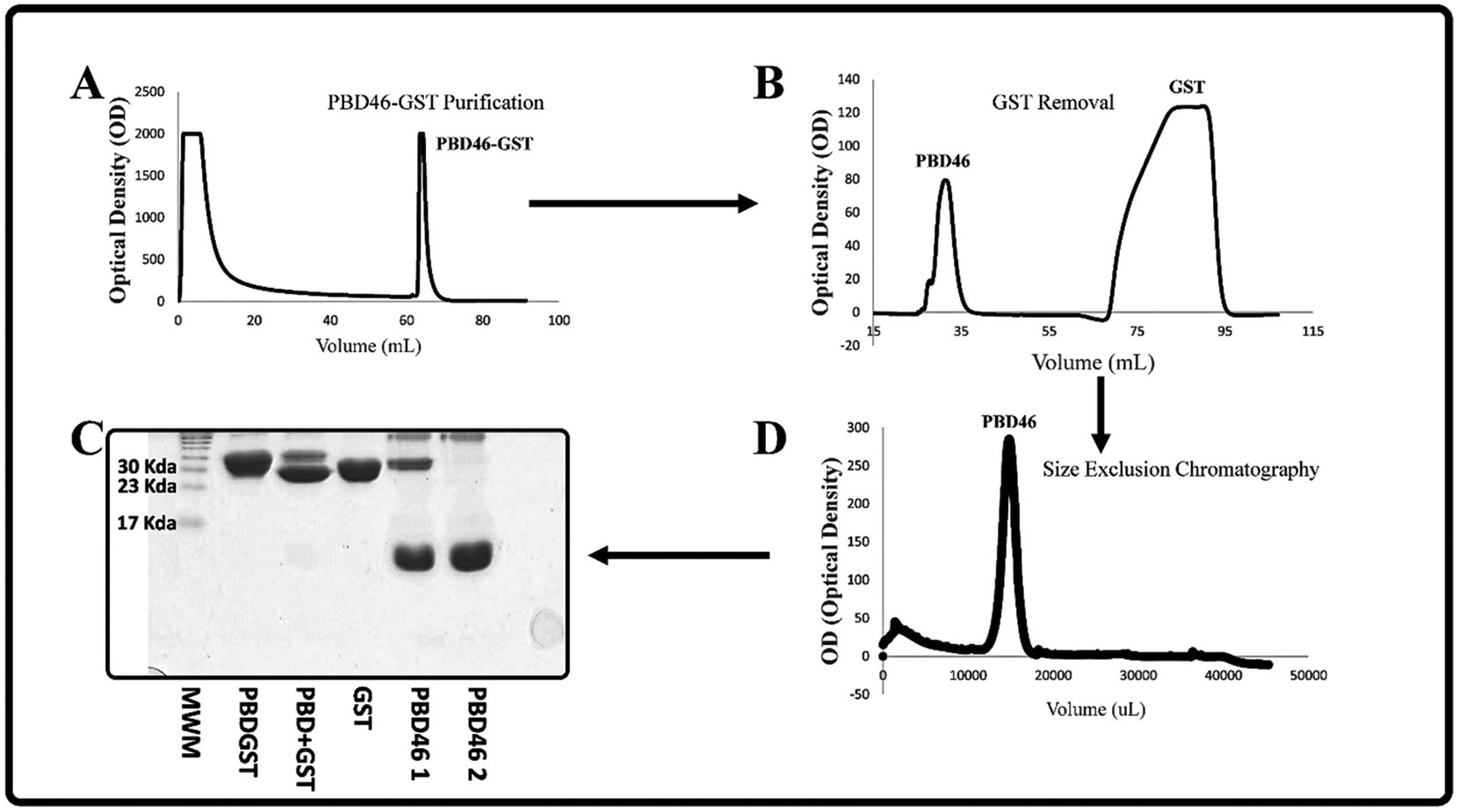
Conventional multi-step purification of PBD46. (A) Glutathione affinity chromatography captures the GST-PBD46 fusion from *E. coli* lysate, shown as a major peak around 70 mL. (B) Thrombin cleavage separates PBD46 from GST, followed by a second glutathione column; distinct peaks correspond to PBD46 (~55 mL) and GST (~90 mL). (C) SDS-PAGE confirms separation: MWM (ladder), PBD-GST, PBD-GST after thrombin cleavage, isolated GST, and two PBD46 samples. (D) Size-exclusion chromatography yields highly pure PBD46 as a single peak.

**Fig. 2.**
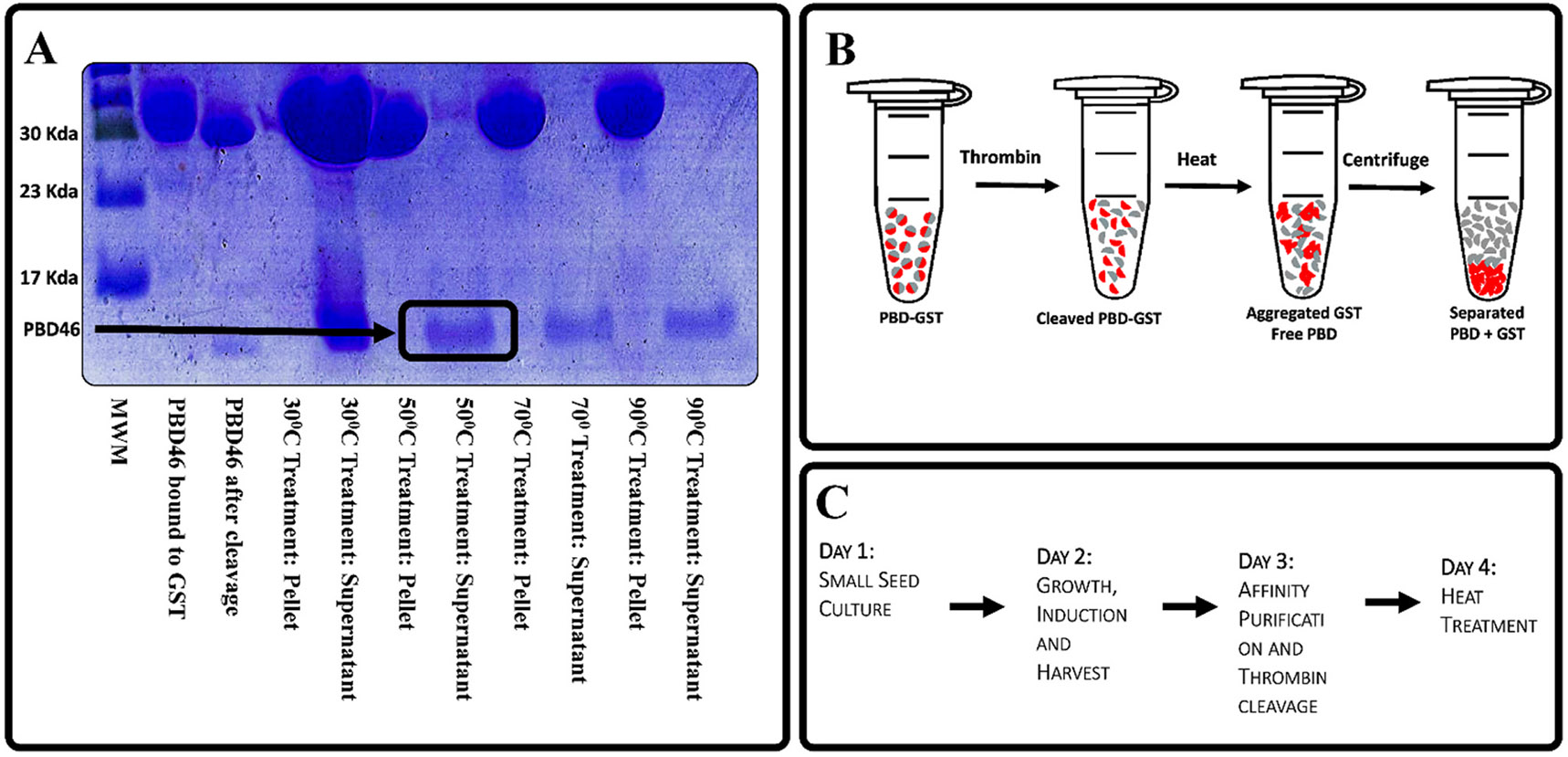
Heat-based one-step purification of PBD46. (A) SDS-PAGE shows supernatant and pellet fractions after heating cleaved samples at 30–90 °C for 30 min. PBD46 remains in the supernatant at ≥50 °C, while GST aggregates into the pellet. (B) Schematic of the workflow: thrombin cleavage, heat denaturation, centrifugation, and collection of soluble PBD46. (C) Summary timeline for the optimized four-day protocol, including culture growth, induction, affinity purification, and heat purification.

**Fig. 3.**
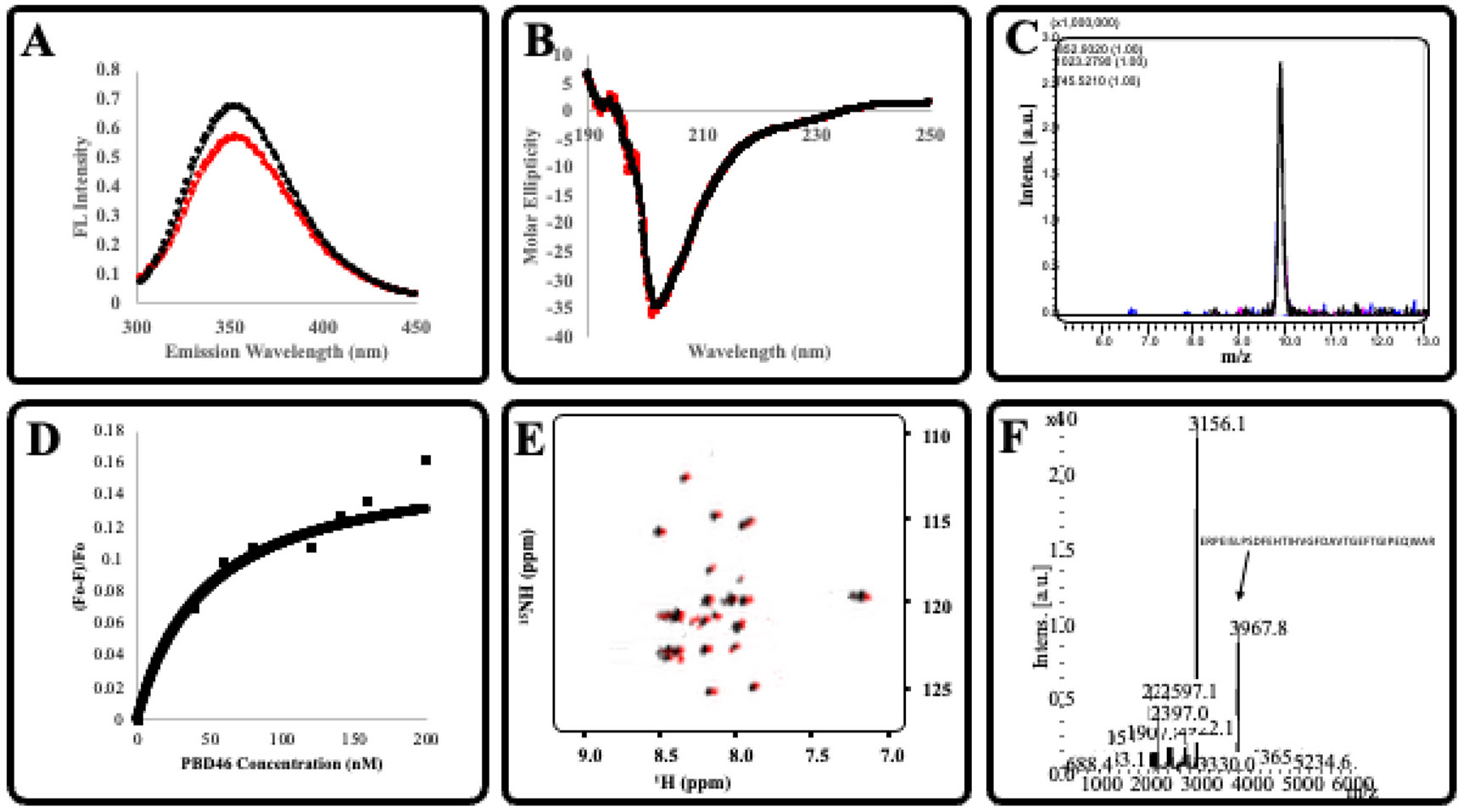
Structural and functional validation of heat-purified PBD46. (A) Intrinsic tryptophan fluorescence spectra (λ_ex_ = 280 nm) of conventional (black) and heat-purified (red) PBD46 are identical. (B) Circular dichroism (CD) spectra (190–250 nm) overlap between both samples, indicating preserved secondary structure. (C) LC-MS analysis shows a sharp peak consistent with the expected molecular weight of PBD46. (D) Extrinsic fluorescence titration using sNBD-labeled Cdc42 yields a binding curve with Kd ≈ 83 nM. (E) 2D ^1^H─^15^N HSQC NMR spectra for ^15^N-labeled PBD46 (heat-purified, red; conventional, black) are overlaid, confirming matched peak positions. (F) MS/MS spectrum identifies peptide fragments covering the full PBD46 sequence.

**Fig. 4.**
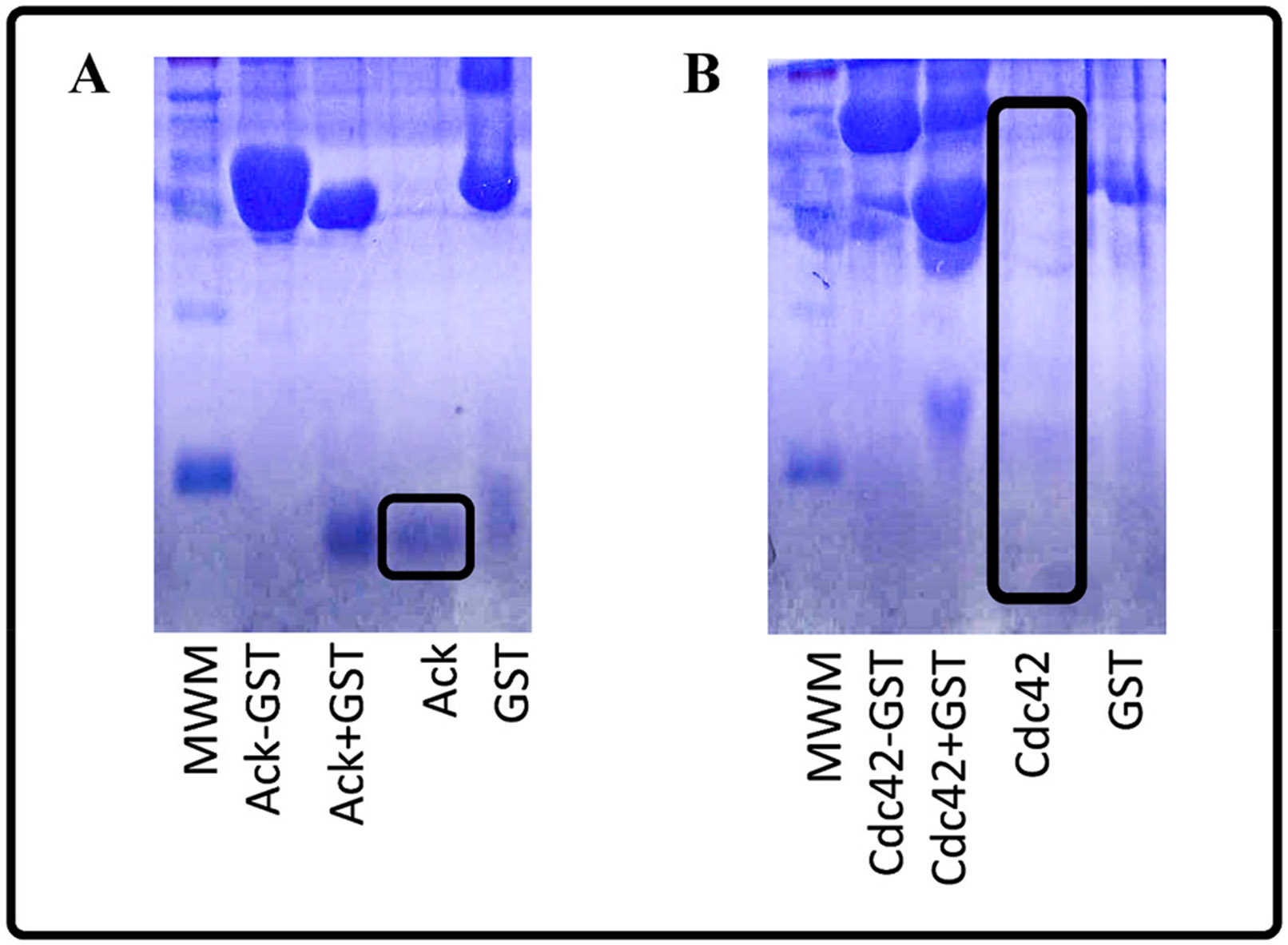
Application of the heat-purification method to other proteins. (A) SDS-PAGE of heat-treated GST–ACK1 fusion ACK1 peptide, ~6 kDa). Lanes: MWM (molecular weight markers), GST–ACK1 before cleavage, GST–ACK1 after cleavage, ACK1 in the Supernatant after heating and centrifugation, GST in the pellet after heating and centrifugation. (B) SDS-PAGE of heat-treated GST–Cdc42 (~21 kDa). Lanes: MWM, GST–Cdc42 before cleavage, GST–Cdc42 after cleavage, supernatant after heating (no Cdc42 observed). These results demonstrate that the one-step heat separation efficiently isolates small, heat-stable peptides (like ACK1 but not larger folded proteins (like Cdc42).

## Data Availability

Data will be made available on request.
